# Host-seeking efficiency can explain population dynamics of the tsetse fly *Glossina morsitans morsitans* in response to host density decline

**DOI:** 10.1371/journal.pntd.0005730

**Published:** 2017-07-03

**Authors:** Jennifer S. Lord, Zinhle Mthombothi, Vitalis K. Lagat, Fatumah Atuhaire, John W. Hargrove

**Affiliations:** 1 Department of Vector Biology, Liverpool School of Tropical Medicine, Liverpool, United Kingdom; 2 SACEMA, University of Stellenbosch, Stellenbosch, South Africa; 3 Department of Mathematical Sciences, University of Stellenbosch, Stellenbosch, South Africa; 4 African Institute for Mathematical Sciences, Muizenberg, South Africa; National Institute of Allergy and Infectious Diseases, UNITED STATES

## Abstract

Females of all blood-feeding arthropod vectors must find and feed on a host in order to produce offspring. For tsetse—vectors of the trypanosomes that cause human and animal African trypanosomiasis—the problem is more extreme, since both sexes feed solely on blood. Host location is thus essential both for survival and reproduction. Host population density should therefore be an important driver of population dynamics for haematophagous insects, and particularly for tsetse, but the role of host density is poorly understood. We investigate the issue using data on changes in numbers of tsetse (*Glossina morsitans morsitans* Westwood) caught during a host elimination experiment in Zimbabwe in the 1960s. During the experiment, numbers of flies caught declined by 95%. We aimed to assess whether models including starvation-dependent mortality could explain observed changes in tsetse numbers as host density declined. An ordinary differential equation model, including starvation-dependent mortality, captured the initial dynamics of the observed tsetse population. However, whereas small numbers of tsetse were caught throughout the host elimination exercise, the modelled population went extinct. Results of a spatially explicit agent-based model suggest that this discrepancy could be explained by immigration of tsetse into the experimental plot. Variation in host density, as a result of natural and anthropogenic factors, may influence tsetse population dynamics in space and time. This has implications for *Trypanosoma brucei rhodesiense* transmission. Increased tsetse mortality as a consequence of low host density may decrease trypanosome transmission, but hungrier flies may be more inclined to bite humans, thereby increasing the risk of transmission to humans. Our model provides a way of exploring the role of host density on tsetse population dynamics and could be incorporated into models of trypanosome transmission dynamics to better understand how spatio-temporal variation in host density impacts trypanosome prevalence in mammalian hosts.

## Author summary

Tsetse flies are the vectors of human and animal African trypanosomiasis. The flies rely solely on vertebrate blood for food and water and changes in vertebrate host density may therefore influence tsetse survival. We develop models including starvation-dependent mortality to explain changes in numbers of the savannah tsetse *Glossina morsitans morsitans* caught during a host elimination experiment in Zimbabwe in the 1960s. Our models provide a way of exploring the role of host density on tsetse population dynamics and could be incorporated into models of trypanosome transmission dynamics to better understand how spatial and/or temporal variation in host density impacts prevalence of trypanosome infection in vertebrate hosts.

## Introduction

For any blood-feeding arthropod vector, the process of finding and feeding on a host is essential for reproduction. Both male and female tsetse flies (Diptera: Glossinidae), vectors of the trypanosomes which cause human and animal African trypanosomiasis, rely exclusively on blood for water and energy. To avoid starvation, a tsetse must find and feed on a host every four to eight days, depending on temperature [1–4]. Females must feed more frequently if they are to produce viable pupae [5].

The probability that a tsetse can find and feed on a host before it starves depends on host density and the fly’s host-seeking efficiency–*i*.*e*., the ability to find and feed on a host given one is present. This depends on the fly’s feeding-related activity levels. It has been variously proposed that: (i) tsetse feed opportunistically [6]; (ii) feeding rates increase linearly or exponentially following each meal [5]; (iii) there is a fixed non-feeding period of about three days after each meal followed by intense activity leading to feeding within 0.3–0.8 days [7,8]. These ideas have not incorporated the impact of host density on the probability of feeding.

Variation in host density may be an important driver of heterogeneity in tsetse population dynamics. Population declines in savanna tsetse, including *Glossina morsitans*, have been observed at the borders of protected areas in East Africa [9,10]. While this decline may be due to a reduction in suitable microclimates in human-dominated landscapes, it may also be due to lower wild host densities resulting from human activities [11]. Indeed, numbers of tsetse caught in traps have, occasionally, been correlated with numbers of hosts in field observations [12,13], and the hosts of savanna tsetse exist at a range of densities. For example, warthog, the host most frequently fed on by *G*. *morsitans* [14–16], are found at densities between 1 and 10 per km^2^ in protected areas (national parks and game reserves), but local densities over 70 per km^2^ have been reported [17].

To our knowledge, no study has focused on the impact of host density, and vector host-seeking efficiency, on the probability of a vector finding a host and, therefore, on vector mortality and reproduction rates. We investigated this issue using data on the changing numbers of hosts and on the numbers of male and female *G*. *morsitans morsitans* Westwood captured during a host elimination experiment in Zimbabwe in the 1960s [18,19]. While host elimination is not usually considered an ethical or sustainable method of tsetse control, the results of existing experimental elimination studies can be used to inform questions about the impact of natural and anthropogenic changes in the density of hosts on tsetse populations.

We therefore aimed to develop mechanistic models of tsetse population dynamics including tsetse host-seeking efficiency and assess their ability to explain the effect of host density decline on observed mean monthly numbers of tsetse caught during a host elimination experiment.

## Methods

### Estimating changes in host density

#### Data

Between October 1962 and September 1967, hunters shot the favoured hosts of *G*. *m*. *morsitans* [14–16], including elephant, buffalo, kudu, bushbuck, warthog and bushpig within a 541 km^2^ area of Nagupande, Zimbabwe (18° 06' S., 27° 22' E.). A game fence surrounded the area to prevent animals re-entering from surrounding regions. The data available [18] include the numbers of hosts shot each month within the 541 km^2^ area. We used these data to estimate the host density in Nagupande before and during the elimination experiment. Whereas these are old data, we used them because they are the only data available that relate the decline in a population of blood-sucking insect to declines in the host population for that insect.

#### Analysis

We assumed that the area within the game fence was closed to migration of the host mammal population, which had a natural growth rate of *r* per month, and that these hosts were shot at constant rate *s* per month. With these assumptions, we applied a removal method of population estimation [19,20] to fit the observed data, the number, *S*(*i*), of hosts shot in month *i* of the study, using:

$$S\left(i\right)=k_{1}\left[exp\left(k_{2}\left(i-1\right)\right)-\text{exp}\left(k_{2}i\right)\right]$$ (1)

where *k*_1_ = *sH*(0)/(*s* − *r*), *k*_2_ = *s* − *r* and *H*(0) is the number of mammals present just before the onset of hunting (see S1 File for full details). In the modelling, we treat *r* as a parameter.

The majority of hosts shot during the experiment were warthog. A maximum rate of increase in warthog densities at Sengwa Research Station, which is 59 km east of Nagupande, was found to be approximately 1.5% per month [21]. We therefore assumed the host growth rate (*r*) to be between zero and 0.015 months^-1^ and compared model fits where we assumed one or other of these two extreme values, or a mean value of 0.007 months^-1^. With an assumed value for *r*, and with the hunting data fitted using Eq 1, *s* and *H*(0) can be estimated from the fitted values of *k*_1_ and *k*_2_. The number, *H*(*t*) of hosts surviving at time *t* after the start of hunting is then given by:

H(t)=H(0)exp(−(s−r)t) (2)

There is anecdotal evidence that hosts quickly become wary and difficult to approach under hunting pressure [22]. This likely accounts for the rapid decline in the numbers shot after the first two months of the experiment [18]. In addition to fitting the model using all months after the start of host elimination (from October 1962, month 10), we also therefore fitted the model excluding data for the first two months (see S1 File for full details). Maximum likelihood was used to fit the model to the data. Given that the catches are integer data and that the catches were small in relation to the total population, it was appropriate to assume that the data were Poisson distributed.

### Explaining tsetse population decline

#### Data

Ten months before the start of shooting, and during host elimination, tsetse flies were sampled daily by walking an ox as bait along six 3000 yard (2743 m) transects and catching any flies attracted to the ox—referred to as a ‘fly-round’. Tsetse were also sampled in the same way at a control site 30 km away at Lusulu (18° 05' S., 27° 50' E.), where no hosts were removed. Catches are expressed as average monthly counts of *G*. *m*. *morsitans* (males and females combined) per 10,000 yards (9144 m) of transect completed [18]. Estimates of the variation in daily catches around the monthly means are not available and we assume the data are Poisson distributed. There are no estimates available of the absolute fly density at Nagupande: we assume that the average monthly catch is proportional to the mean population in the area during that month.

#### Models

To model the changes in tsetse density before and during the host elimination experiment, we developed an ordinary differential equation (ODE) model, assuming a closed system with no immigration or emigration of flies. We also developed a spatially explicit agent-based model (ABM), to compare model fits to the data with and without the assumption of in- or out-migration of tsetse.

#### ODE model description

**Fig 1:**
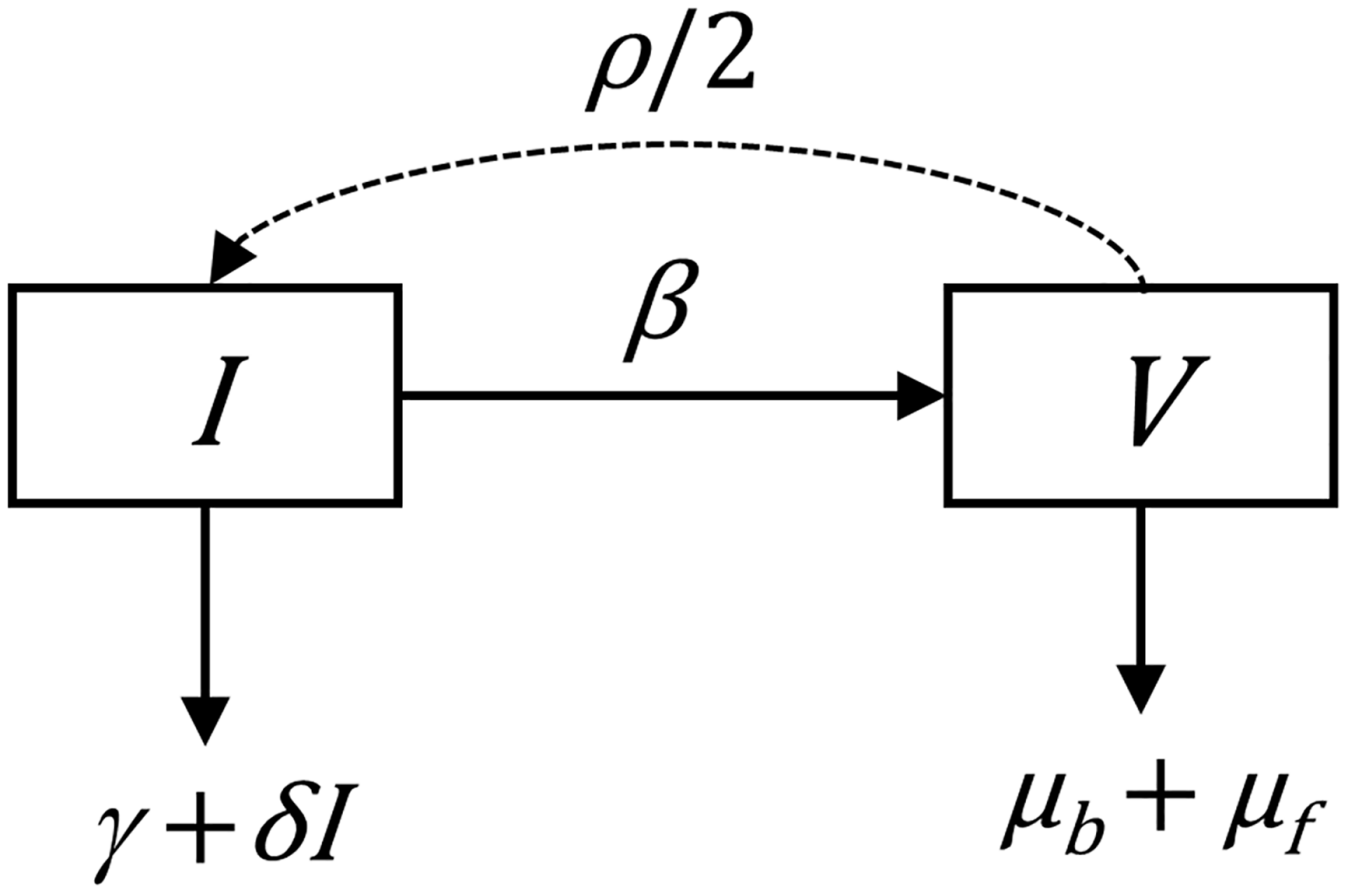
Ordinary differential equation model of tsetse population dynamics. Pupae, *I*, metamorphose into adults, *V* at rate *β*. We take *ρ* as the rate of production of pupae by adult females. Only half of the emerging flies are female, so female pupae are produced at rate *ρ* /2. Adult losses are due to background mortality at rate *μ*_*b*_ and starvation mortality at rate *μ*_*f*_, both considered density independent. Pupal losses are divided between density-independent mortality *γ*, and density-dependent mortality with coefficient *δ*. All rates have units days^-1^.

Tsetse essentially have three life stages—adult, larva and pupa—but the post-larviposition, and pre-pupariation, larval stage is brief relative to the other stages. Accordingly, our ODE model consists of two ordinary differential equations to model population densities of adults and pupae (both males and females) over time (Fig 1).

As for any species, tsetse population regulation in the long term must involve density-dependent changes in at least one of the birth, death or migration processes. In tsetse, the process is poorly understood [23–25]. For simplicity, we assume that density-dependent mortality acts only at the pupal stage [23].

To incorporate the effects of host density on tsetse mortality rates, we partition the adult daily mortality rate between a starvation mortality rate (*μ*_*f*_) that varies with host density and a background mortality (*μ*_*b*_) including deaths from other causes including senescence and predation. The starvation mortality rate will approach zero in situations where there is a high host density.

With the above definitions and assumptions, we represent changes in the density of immature tsetse *I* and adult vectors *V* over time by the following ordinary differential equations:

$$\frac{dI}{dt}=\rho \;V/2-\left(\beta +\gamma +\delta I\right)I$$ (3)

$$\frac{dV}{dt}=\beta I-\left(\mu_{b}+\mu_{f}\right)V$$ (4)

In S2 File we show that if we have *H* hosts in an area of *A* sq km, and set λ = *H/A*, the probability, *F’*(*λ*, *σ*, *ν*), a fly *fails to feed* on *v* consecutive days, and hence starves, is:

$$F^{\prime }\left(\lambda ,\sigma ,\nu \right)=\text{exp}\left(-\lambda \sigma \nu \right)$$ (5)

where *σ* is the daily probability of finding and feeding on a host, given one host present within a square area, of side 1 km, of the fly. The daily mortality due to starvation is then:

$$\mu_{f}=-(\text{ln}\left(1-\text{exp}\left(-\lambda \sigma \nu \right)\right))/\nu$$ (6)

The ODE model in Eqs 3 and 4 implies an exponentially distributed pupal period with, therefore, a non-zero probability that adults start emerging from puparia immediately after larviposition. In reality, where the mean pupal duration always exceeds 20 days, no adults start emerging sooner than about 3–4 days prior to this mean duration. We model this process more realistically using an Erlang distribution with shape parameter *n* and scale parameter *nβ*. This results in *n* sub-compartments within the *I* stage of the ODE model and a mean rate of emergence as adults equivalent to that in the exponentially distributed model.

#### ODE model fitting

The model in Eqs 3 and 4 was fitted to the Nagupande *G*. *m*. *morsitans* data. The data used to fit the model consisted of the observed monthly mean catches of tsetse over days 1–285, before hunters began shooting hosts, and over days 286–2055 while hosts were being shot. Using tsetse counts before and during host elimination allowed us to distinguish between background and starvation-dependent mortality, fitting both *σ* and *μ*_*b*_.

We assume that the average monthly catch is proportional to the mean population in the area during that month. The initial number of adult tsetse, at *t* = 0, was accordingly set at 250 –approximately the number caught in the first month of sampling. Parameters other than *σ* and *μ*_*b*_, were taken from the literature where possible (Table 1): we assumed a mean temperature of 20°C, reflective of the average temperature at Nagupande [26]. We fitted the model to the data using maximum likelihood, assuming the data were Poisson distributed. We compared model fits for runs where both *σ* and *μ*_*b*,_ were fitted, to fits where we assumed *σ* = 1, such that there was no starvation-dependent mortality.

**Table 1:** Model parameter inputs.

Parameter	Symbol	Value (range)	Source
Female adult larviposition rate	*ρ*	1/11* (1/8–1/12) days^-1^	[44,45]
Adult emergence rate	*β*	1/45* (1/30–1/50) days^-1^	[26,45]
Pupal density-independent mortality rate	*γ*	0.006* (0.0025–0.01) days^-1^	[23,26]
Pupal density-dependent mortality coefficient	*δ*	0 (0–0.01)	N.A.
Average time to starvation	*v*	6* (4–8) days	[1–4]
Host growth rate	*r*	0.007 (0–0.015) months^-1^	[21]

*Estimated from the literature assuming a temperature of 20°C.

As we do not know the exact values for the input parameters, we quantified how changes in parameter inputs affected model fits and estimates of *σ* and *μ*_*b*,_ by fitting the model multiple times, using all possible combinations of the assumed and extreme values of *ρ*, *β*, *γ*, and *v* (Table 1). As there are no estimates of *δ* in the literature, we used five different values for this parameter—0, 0.00001, 0.0001, 0.001 and 0.01 in the sensitivity analysis. In addition, as we do not know the initial density of pupae, we also fitted the model assuming different starting densities of 50, 250 and 500 for each combination of other parameter values. Fits resulting in *μ*_*b*_ less than 0.005 were omitted, since values lower than this imply biologically unfeasible average survival times of 200 days for adult tsetse [27]. Using the model fits from runs with each combination of input parameter values, we assessed the effect of each input parameter on fitted values of *σ* using partial rank correlation coefficient (PRCC). The PRCC provides a measure of the strength of the relationship between an input variable and an output after the removal of the effects of all other input variables [28].

In addition to fitting the ODE model as described in Eqs 3 and 4, we fitted the model to the data with the assumption that the pupal period is Erlang distributed. To do this we assumed *n* to be 20, the mean adult emergence rate to be equivalent to *β* and used *δ* = 0.00001, with all other values as per Table 1. The fitted parameter values of *σ* and *μ*_*b*_ from the Erlang distributed model were used as parameter estimates within the ABM.

#### Spatial model

We used a spatially explicit ABM to compare changes in numbers of tsetse during the experiment between the closed-system scenario and a scenario allowing tsetse movement into and out of the experimental plot. The model, developed in Netlogo [29], is fully described in S3 File.

We did not model, explicitly, changes in numbers of individual hosts. Instead, we used Eq 2 to calculate the number of hosts within the experimental plot at each time step, with parameter estimates obtained from model fits to the number of hosts shot each month, as described for the ODE model. For the scenario allowing tsetse movement into and out of the experimental plot, we assumed the density of hosts outside the experimental plot to be equal to that inside, before the start of host elimination.

Except for adult movement, the input parameter values for the ABM reflected those of the ODE (Table 1). In the ABM, however, female adult flies produce a pupa exactly every 1/*ρ* days and pupae become either adult males or females with equal probability after exactly 1/*β* days. This is a fundamental difference between the ODE model and ABM. In the ABM there is zero probability that females can produce a larva before 1/*ρ* days or that pupae can emerge as adults before 1/*β* days. In the ABM, during each time step of one day, adult males and females move a total straight-line distance of 0.5 cells in a random direction [30].

We carried out ten replicate model runs using the fitted values of *σ* and *μ*_*b*_ obtained from the ODE model fit, assuming an Erlang distributed pupal period with *n* = 20 and scale *nβ*, *δ* = 0.00001 and all other parameters as per Table 1. For the closed-system scenario, we recorded total numbers of adult tsetse each day. For the open-system scenario, we recorded numbers of adult tsetse present in the experimental plot. Simulated counts from replicates were averaged and log(*x* + 1) transformed and then the residual sum of squares (RSS) was used to compare closed and open simulation results to the log(*x* + 1) transformed observed data. As a check of model outputs, we compared the carrying capacity, and ratio of pupae to adults at carrying capacity, for the closed scenario ABM with the corresponding ODE outputs, assuming an Erlang distributed pupal period, and assuming no host decline in both models.

## Results

### Changes in host density

**Fig 2:**
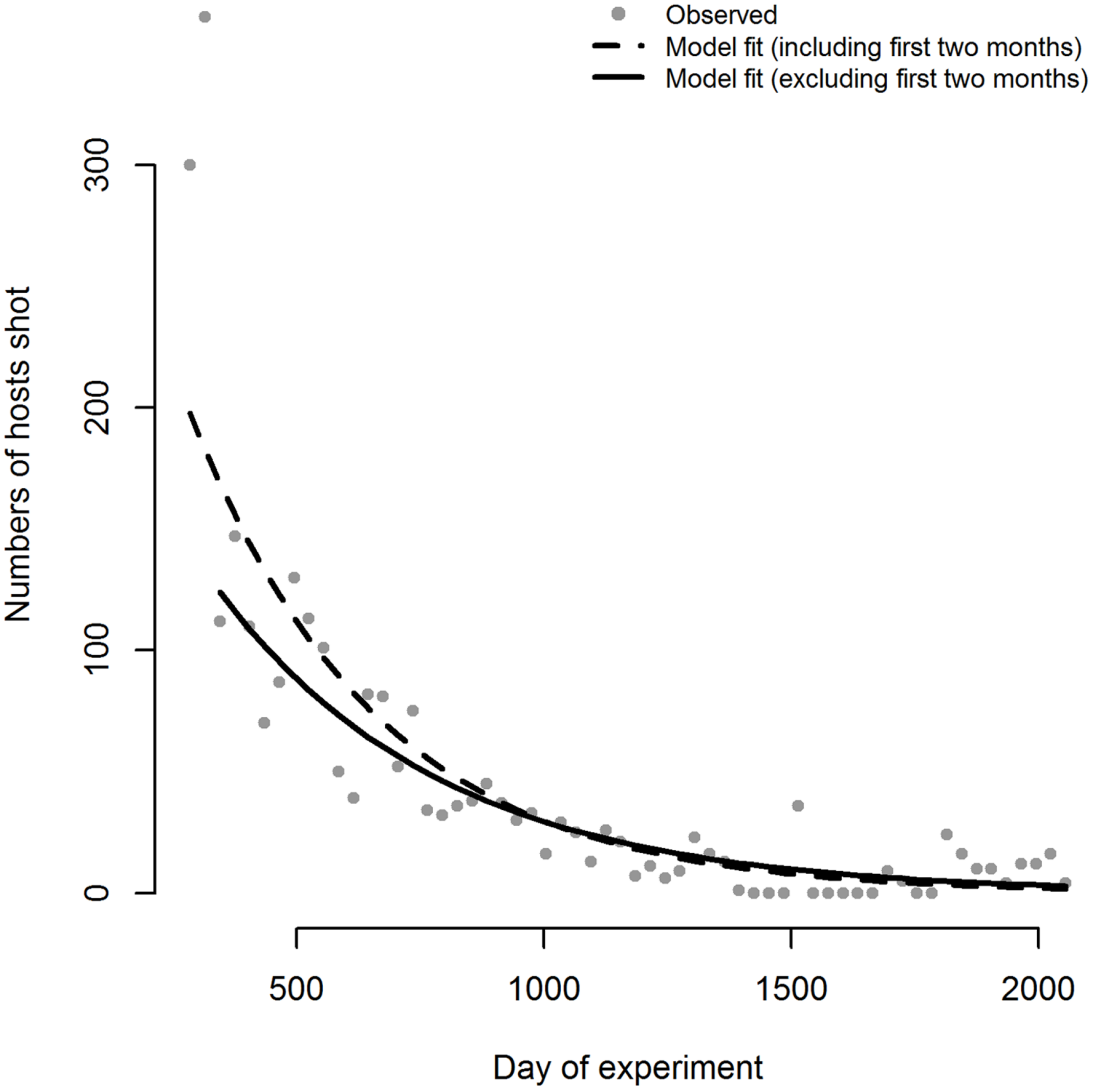
Numbers of hosts shot during the Nagupande experiment. The numbers shot each month are modelled using Eq 1 –either modelling all data together with a constant kill rate throughout (dashed line), or assuming that the kill rate was higher during the first two months of the study (solid line). Assuming a growth rate (*r*) of 0.007 month^-1^ the model fitted to data for all months gave estimates of *k*_*1*_ = 2591 (2492–2693), *k*_*2*_ = 0.0794 (0.0027–0.0828). Excluding the first two months gave estimates of *k*_*1*_ = 1951 (1865–2041), *k*_*2*_ = 0.0657 (0.0621–0.0693) for month 12 onwards.

**Fig 3:**
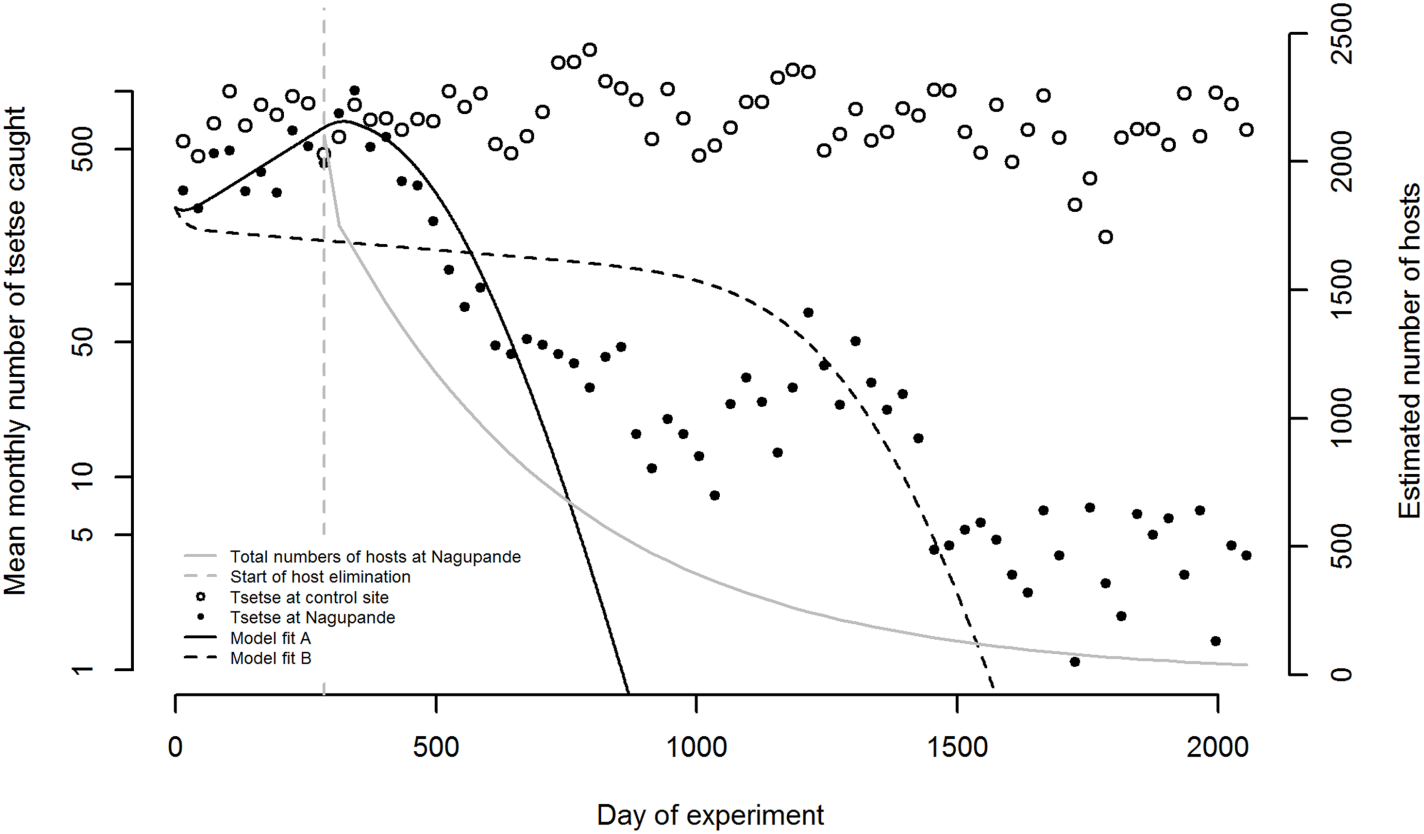
Ordinary differential equation (ODE) model fits to the mean monthly number of tsetse caught during ox-baited fly-round, and estimated number of hosts during the Nagupande experiment. Host numbers assumed constant prior to the start of hunting. Mean monthly catches of *G*. *m*. *morsitans* in the Nagupande area (closed circles) and at the control site in Lusulu (open circles) are plotted on a logarithmic scale. Model fit A: fitting both *σ* (daily probability of finding and feeding on a host, given one host present within a square of side 1 km area around the fly), and *μ*_*b*_ (adult background mortality rate). *σ* = 0.124 (0.123–0.125); *μ*_*b*_ = 0.0180 (0.0176–0.0183) days^-1^; log likelihood 2216. Model fit B: assuming no mortality due to starvation (*σ* = 1) and fitting only background mortality *μ*_*b*_, 0.03697 (0.03696–0.03699) days^-1^; log likelihood 4694. Fits use the values for input parameters given in Table 1. Note that we here assume no movement of flies into and out of the experimental plot.

We fitted the model in Eq 1 to the data on the numbers of hosts shot each month (Fig 2) and used the resulting values of *k*_1_ and *k*_2_ to estimate *H*(0), the number of hosts present just before hunting commenced. With an assumed host growth rate of 0.007 month^-1^ and using values of *k*_*1*_ and *k*_*2*_ from fitting the model to data from all months, we estimated that there were 2380 hosts within the 541 km^2^ area of Nagupande, prior to the start of host elimination. The estimated figures were 2591 and 2179 for assumed growth rates of 0 and 0.015 month^-1^, respectively (S4 File). Assuming a growth rate of 0.007 month^-1^, but fitting the model to the data excluding the first two months, resulted in an estimate of *H*(*0*) of 1763 at the start of month 12. Then calculating the numbers of hosts present during months 10 and 11 separately, accounting for the numbers killed each month and the growth rate, gave an estimated 2100 hosts before the start of host elimination. Fitting the first two months separately, we estimated that the initial hunting rate was 0.13 month^-1^, and was 0.066 month^-1^ from month 12 onwards. This led to a decline in host density from approximately four hosts per km^2^ at the start of the experiment, to <0.1 hosts / km^2^ by the end of the experiment (day 2055) (Fig 3). We use this estimation for the analysis of changes in tsetse density over time.

### Explaining tsetse population decline

The average numbers of tsetse caught in Nagupande per month increased during the first two months of host elimination, but then declined from approximately 1000 per month to fewer than 50 by month 20 (day 600). After day 600, the rate of decline decreased, with an average of one to five flies still being caught per month after day 1500. In contrast, at the control site, mean tsetse catches remained above 500 for most of the experiment (Fig 3).

For the initial part of the experiment, up to day 600, adding starvation-dependent mortality to the ODE model—(model fit A, Fig 3) given assumed fixed parameter inputs (Table 1)—improves the fit to the data (model fit B, Fig 3). Both models predicted, however, that the tsetse population went extinct, whereas tsetse were caught in Nagupande up to the end of the experiment, albeit in smaller numbers than at the control site.

Given the assumed input parameters in Table 1, the fitted value of *σ* was 0.124 (0.122–0.126) and the fitted value of *μ*_*b*_ was 0.018 (0.017–0.019) days^-1^. According to our model of starvation-dependent mortality (Eq 6), these fitted values, and the estimated changes in host density during the experiment, the starvation-dependent mortality rate of adult flies started at less than 0.01 days^-1^ and increased non-linearly, by a factor of about 50, to approximately 0.5 days^-1^ by the end of the experiment.

Model runs varying the values of the input parameters according to the ranges given in Table 1, and resulting PRCC analysis, indicated that days to starvation (*v*) and the pupal density-dependent mortality coefficient (*δ*) had a strong influence (PRCC greater than +/- 0.5) on values of *σ*. An increase in the number of days to starvation led to a decrease in the estimate of *σ*: increases in the pupal density-dependent mortality coefficient resulted in increases in the estimate of *σ*.

Given the minimum expected value of *v*, of four days to starvation, fitted estimates were *σ* = 0.241 (0.240–0.242) and *μ*_*b*_ = 0.0217 (0.0215–0.0220). If flies only starved after eight days, *σ* = 0.0769 (0.0768–0.0773) and *μ*_*b*_ = 0.0156 (0.0152–0.0157). Similarly, taking *δ* = 0.0001 resulted in estimates of *σ* = 0.1873 (0.1871–0.1878) and *μ*_*b*_ = 0.0112 (0.0111–0.0113), whereas with *δ* = 0.001, *σ* = 0.1879 (0.1877–0.1892) and *μ*_*b*_ = 0.00137 (0.00136–0.00147). For *δ* = 0.01 adult background mortality rates were less than 0.001 days^-1^.

Using the Erlang distributed pupal period ODE model with shape *n* = 20, scale = *nβ* and *δ* = 0.00001 resulted in fitted values of *σ* = 0.1350 (0.1347–0.1359) and *μ*_*b*_ = 0.0113 (0.0109–0.0116) (log likelihood 2150). These estimates were within the range of estimates obtained using the ODE with exponentially distributed pupal period during sensitivity analysis. We used the fitted values from the Erlang distributed model as parameter estimates in the ABM.

**Fig 4:**
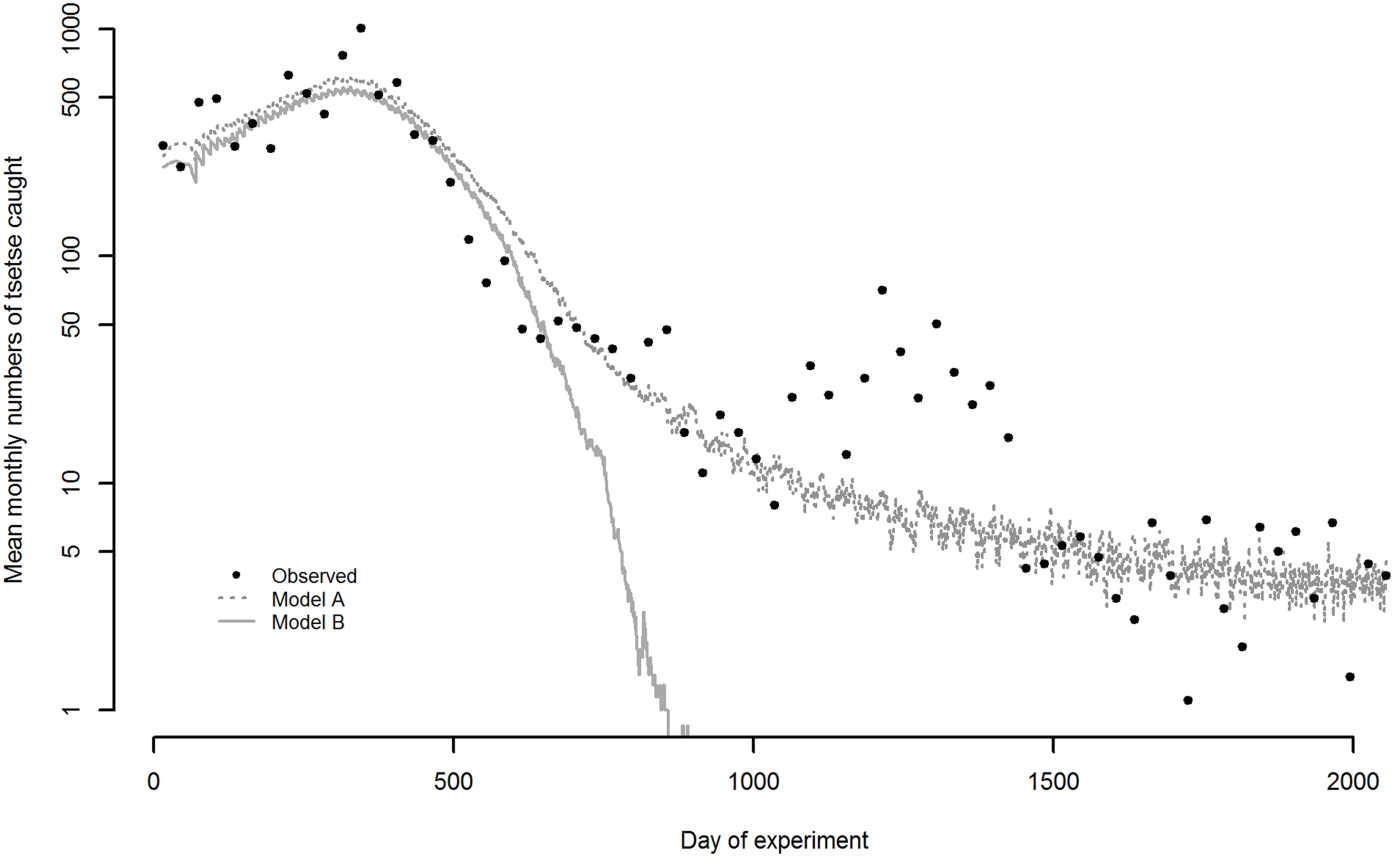
Output from agent-based model (ABM) of tsetse population dynamics including starvation-dependent mortality. Assuming *δ* = 0.00001, *σ* = 0.135 and *μ*_*b*_ = 0.011. Lines represent the means over 10 model runs. Model A: assuming movement of flies into and out of the experimental area, with flies moving a straight-line distance of 0.5 cells/ 250m per day in a random direction, sum of squared residuals– 43. Model B: assuming a closed system with no movement of flies into or out of the experimental area. Sum of squared residuals– 294. *σ* is the daily probability that a fly finds, and feeds on a host, given one host is present within a square of side 1 km around the fly.

The ABM allowing for fly movement into and out of Nagupande from surrounding areas provided a better fit to the data than the scenario assuming a closed system, with flies still present after day 1500 (Fig 4A). Assuming a closed system, reflecting the assumption in the ODE that there was no movement of flies into or out of Nagupande, the ABM simulated fly population went extinct before day 1000 (Fig 4B) as also occurred according to the ODE model results (Fig 3A).

The ODE model simulations without host decline resulted in ~ 1.7 adults to every pupa assuming the pupal period was exponentially distributed, but an Erlang distribution with *n* = 20 resulted in ~ 1.1 pupae to every adult at carrying capacity. This compares with ~ 1.2 pupae to every adult in the ABM.

## Discussion

Our models of tsetse population dynamics, incorporating vector host-seeking efficiency and movement of flies into and out of the Nagupande experimental area, provided good fits to the observed decline in catches of *G*. *m*. *morsitans* after reductions in host numbers. After day 600, when host density had been reduced by about 50%, mean monthly numbers of flies caught declined to less than 5% of the maximum numbers seen before host elimination. Catches never went to zero, however, consistent with the idea that there was immigration into the experimental area. Predicted daily catches in the experimental area varied from 0 to 5% of the peak levels, also consistent with a small flow of immigrant flies. Variation in catches late in the experiment could also have been influenced by: (i) a temporary increase in fly numbers following the introduction, in February 1965 (around day 1125), of 90 head of cattle into the Nagupande experimental area [18]; (ii) the initiation, in October 1964 (around day 1000) of host elimination in areas surrounding Nagupande. We did not account for these factors in our model.

In a similar experiment carried out at Sengwa Wildlife Research Area (18° 10' S., 28° 13' E.), approximately 30 km from Nagupande, there was no discernible effect of host removal on the numbers of tsetse caught over time [31]. The experimental area was, however, only 10.9 km^2^, and we suggest that, in this case immigration of flies into the area was sufficient to mask the effect of host reduction.

### Feeding models

Laboratory experiments show that *G*. *m*. *morsitans* becomes more active with time since last blood-meal [32], and feeding rates will likely increase with days since last feed, as suggested in various tsetse feeding models [5,6,33,34]. We do not attempt to decide between these models; our estimate that a fly has a 10–20% chance of finding and feeding on a host in one day, given one host present within a square area, of side 1 km, of the fly, is simply an average estimate across all days of the feeding cycle. The estimate contrasts strongly with estimates that an odour-baited insecticide-treated target kills less than 1% per day of *G*. *morsitans* within a 1 km^2^ neighbourhood [35]. The greater ability of flies to find live hosts is attributable in part to the fact that odour-baited targets lack some of the odour components (particularly carbon dioxide) present in live hosts. Moreover, real hosts are mobile and thus provide increased opportunities for flies to find, and feed on, them if they walk past the point where a tsetse fly is resting. This latter point may be particularly important for *G*. *m*. *morsitan*s, since this savannah species appears to adopt a predominantly ‘sit-and-wait’ approach to host-seeking [36].

### Limitations of the study

We have ascribed declines in tsetse numbers at Nagupande to increased levels of starvation consequent on the reduced probability that flies locate a host as host numbers declined. We have not allowed for the possibility that a reduction in host numbers results in an increase in the vector-to-host ratio and a density-dependent reduction in feeding success for flies that have located a host [23–25]. Similarly, a reduction in numbers of preferred hosts could lead flies to feed in riskier situations, or on more dangerous hosts, such as humans and baboons. All such effects would, however, result in increases in adult mortality: it was thus unnecessary to stipulate the precise way in which feeding-related mortality increased in order to capture the dynamics of the situation.

Our models assume that females are inseminated at emergence. This may be a suitable assumption for large population sizes but, as the population declines toward extinction, the probability increases that a female will fail to find and mate with a male fly. This simplifying assumption may have affected our parameter estimates obtained from fitted models, but it does not detract from the ability of our model to explain the observed results.

More complex versions of our model may consider the possibility that reduced host densities are liable to affect tsetse birth rates even more seriously than they affect mortality. Thus, while we set the minimum value of *ν* (time to starvation) to four days, female flies need to feed every 2.5–3 days if they are to produce viable pupae [5]. This requirement puts stricter constraints on the feeding behaviour of female tsetse and means that the required value of search efficiency *σ* will be higher for female flies than estimated here. It was difficult to investigate these more nuanced models, however, since the tsetse catch data available to us consisted only of the total number of flies captured, without providing details of the numbers of each gender or the reproductive state of females.

Whereas density-dependent effects are essential for the long-term stability of any biological population, the addition of density-dependent pupal mortality did not improve model fits to the data. This may be because the population, before and during the experiment, was below the threshold at which density dependent mortality becomes a significant factor, or our model was not set up in such a way as to be able to detect density-dependent effects. Other models of tsetse population dynamics in Zimbabwe, similarly and perhaps for the same reasons, failed to demonstrate density-dependent mortality [37].

### Host density and tsetse population dynamics

Regardless of the mechanisms involved, the Nagupande experiment demonstrates that changes in host density can drive changes in tsetse populations. This may be an important factor in natural situations where densities of warthog, buffalo, elephant, giraffe [16,38], commonly fed on by savannah tsetse, can vary between years from over 20 per km^2^ to fewer than 5 per km^2^ in a single location [39]. Moreover, as one moves from protected areas into farmland, host densities may drop below those required for sustaining tsetse populations.

Densities of wild and domestic hosts are, similarly, essential components in models of trypanosome transmission [40,41], but the effects of varying host density on transmission dynamics have not been explicitly studied. In addition to the effects of host density on vector density, and mortality rates, the existence of hungrier flies in regions where wild hosts are scarce can have implications for trypanosome transmission. First, hungrier flies may be more inclined to bite less-favoured hosts, including humans, thereby increasing the risk of transmission of the zoonotic Rhodesian human African trypanosomiasis [42]. Second, laboratory experiments suggest that starved flies are more susceptible to acquiring a trypanosome infection [43], which may influence transmission dynamics.

Our model provides a way of exploring the role of host density on tsetse population dynamics, predicting where tsetse populations are likely to be highest and where control efforts will be required, and establishing the role livestock may play in supplementing low wild host densities. The model could also be incorporated into models of trypanosome transmission dynamics to better understand how spatial and/or temporal variation in host density impacts prevalence of trypanosome infection in reservoir and target host species.

## Supporting information

S1 File Model to estimate changes in host population density during the Nagupande game elimination experiment. (DOCX)

S2 File Host location probability, and probability of starvation for tsetse, as a function of host density and search efficiency. (DOCX)

S3 File Agent-based model description. (DOCX)

S4 File Effect of host growth rate on estimated numbers of hosts during Nagupande experiment according to model of host kills over time (excluding the first two months). (DOCX)
