# Supplementary material for: Host-seeking efficiency can explain population dynamics of the tsetse fly Glossina morsitans morsitans in response to host density decline
Source: PLoS Negl Trop Dis. 2017 Jul 3;11(7):e0005730. doi: 10.1371/journal.pntd.0005730 (PMC5510883; doi:10.1371/journal.pntd.0005730)
Supplement: S1 File — (DOCX) [file pntd.0005730.s001.docx]

**Supplementary File 1. Model to estimate changes in host population density during the Nagupande game elimination experiment**

We assume that host births, and deaths not due to hunting, occur at constant rates to result in a growth rate *r*. If hosts were shot at rate *s_,_* then the change in the number of hosts (*H*) over time is defined by:

${dH(t)}/{dt}=\left( r-s \right)H(t)$

With solution$H(t)= H(0)\exp\left( \left( r-s \right)t \right) (S1.A)$

The number, *S*(*i*), of hosts shot during month *i*:

$$S\left( i \right)= \int_{i-1}^{i} sH(0)\exp(\left( r-s \right)t)dt$$

$$= k_{1}\left[ exp\left( k_{2}\left( i-1 \right) \right)-\exp\left( k_{2}i \right) \right] \left( S1.B \right)$$

where $H(0)$is the number of hosts before the start of the experiment, $k_{1}= {-sH(0)}/{(s-r)}$ and$k_{2}=r-s$. If we assume a value for *r*, then *s* and $H(0)$can be estimated from *k_1_* and *k_2_* and the number of hosts remaining in the area at any time during the experiment can be estimated.

In addition to fitting the model and estimating the rate at which hosts were shot using all months (from October 1962, month 10) we also fitted the model excluding the first two months. This gave a rate at which hosts were shot from month 12 onwards. To calculate the numbers of hosts in month 11, the numbers shot in month 12 were added to the estimate of numbers of hosts at the start of month 12, adjusting for a growth rate of 0.007 months^-1^. The same was done to calculate the numbers of hosts present in months 9 and 10 – accounting for numbers at the start, numbers shot, and the growth rate. With the estimated numbers of hosts for months nine to 12, *s* was estimated separately for this period by fitting the model of numbers of hosts over time (Equation S1.B) to the numbers of hosts for months 10, 11 and 12, with *H*(0) at time zero equalling the estimated numbers at month 9.
