# Supplementary material for: Host-seeking efficiency can explain population dynamics of the tsetse fly Glossina morsitans morsitans in response to host density decline
Source: PLoS Negl Trop Dis. 2017 Jul 3;11(7):e0005730. doi: 10.1371/journal.pntd.0005730 (PMC5510883; doi:10.1371/journal.pntd.0005730)
Supplement: S2 File — (DOCX) [file pntd.0005730.s002.docx]

**Supplementary File 2. Host location probability, and probability of starvation for tsetse, as a function of host density and search efficiency.**

We define:

*A* Total area (km^2^), assumed closed to host immigration and emigration.

*H* Total hosts, assumed randomly distributed in area A.

We set λ = *H*/*A*

*P_k_* Probability there are k hosts resident, on a given day, in a given

‘neighbourhood’ (defined as a square area, of side 1 km, around the fly).

*i* Number of days since the fly last fed.

*σ* Conditional probability that a fly finds a host and feeds on a given day, given

that it is the only host resident in a 1 km^2^ neighbourhood containing the fly,

and given that the fly is active.

*ν* Maximum number of days fly can survive without feeding.

*f_i_* Probability that a fly succeeds in feeding on day *i* after its last blood-meal.

*F*(λ, σ, ν) Probability that a fly succeeds in feeding at some point in a given hunger cycle of duration *ν* days, given host density *λ* and search efficiency *σ*.

The complement of a probability is denoted by a ‘prime’ suffix; e. g. *f_i_* ′= 1 − *f_i_* .

On any particular day, the probability that a given animal is present in a given 1 sq km neighbourhood of a given fly is 1/*A*. If, as in the Nagupande experiment, *A* is large, then 1/*A* is small. Assuming independence between the hosts in their movement, the probability (*P_k_*) that there are *k* animals present in this neighbourhood can then be defined approximately by a Poisson distribution with parameter *λ* = *H*/*A*. That is:

$P_{k}=\frac{\lambda^{k}}{k!}\exp(-\lambda)$ (S2.A)

If there is only one host in the fly’s neighbourhood the probability that the fly fails to feed off it on a given day, given that the fly is active, is *σ*′. If there are *k* hosts in the neighbourhood and we assume independence among the hosts, then the fly fails to feed off any of them with probability (*σ*′)*^k^*. The probability that it *does* feed, given that there are *k* hosts present and given that the fly is active, is thus 1 - (*σ*′)*^k^*.

With the above definitions, the probability (*f_i_*) that a fly succeeds in feeding on day *i* of its hunger cycle is:

$\begin{matrix} f_{i}=\sum_{k=1}^{\infty} (1-(\sigma')^{k})P_{k} \\ =\exp(-\lambda)\sum_{k=1}^{\infty} (1-(\sigma')^{k})\frac{\lambda^{k}}{k!} \\ =\exp(-\lambda)[\sum_{k=1}^{\infty} \frac{\lambda^{k}}{k!}-\sum_{k=1}^{\infty} \frac{(\sigma'\lambda)^{k}}{k!}] \\ =\exp(-\lambda)(\exp(\lambda)-\exp(\lambda\sigma')) \\ =1-\exp(-\lambda(1-\sigma^{'})) \\ =1-\exp(-\lambda\sigma) \end{matrix}$ (S2.B)

The probability *F’*(*λ,σ,ν*) that, given search efficiency *σ* and host density *λ* = *N*/*A*, the fly *fails to feed* on *ν* consecutive days, and hence starves, is:

$\begin{matrix} F'(\lambda,\sigma,\nu)=\prod_{i=1}^{\nu} (1-f_{i}) \\ =\prod_{i=1}^{\nu} \exp(-\lambda\sigma) \\ =\exp(-\lambda\sigma\nu) \end{matrix}$ (S2.C)

If the daily mortality due to starvation is *μ_f_* then a fly escapes starvation on ν consecutive days with probability exp(-*νμ_f_*)and it follows that

$$\begin{matrix} \exp(-\nu\mu_{f})=1-F'(\lambda,\sigma,\nu) \\ =1-\exp(-\lambda\sigma\nu) \end{matrix}$$

and $\mu_{f}=-(\ln(1-\exp(-\lambda\sigma\nu)))/\nu$ (S2.D)
