# Supplementary material for: Host-seeking efficiency can explain population dynamics of the tsetse fly Glossina morsitans morsitans in response to host density decline
Source: PLoS Negl Trop Dis. 2017 Jul 3;11(7):e0005730. doi: 10.1371/journal.pntd.0005730 (PMC5510883; doi:10.1371/journal.pntd.0005730)
Supplement: S3 File — (DOCX) [file pntd.0005730.s003.docx]

**Supplementary File 3. Agent-based model description**

*Agents, state variables and scales*

There are two options for the spatial extent of the model, which is comprised of patches in a grid. Each patch is equivalent to a square of side 500 m. The first option is a ‘closed’ system where only the Nagupande experimental plot is modelled. This consists of a grid of 90 by 24 patches – equivalent to 540 km^2^. The second option is an ‘open’ system, which includes the areas surrounding Nagupande, consisting of a 150 by 80 cell rectangle – equivalent to 3000 km^2^. Within this area, there is an inner rectangle of 90 by 24 cells (540 km^2^) representing the experimental plot. For both scenarios, the model has reflective boundaries. The model runs for a total of 2055 days, with each time step (tick) representing a single day.

*Global variables set via the interface*

- Model-host-decline: if ‘true’ the model includes host decline, if ‘false’ the number of hosts stays constant.
- Closed-system: if ‘true’, only the experimental plot is modelled, if ‘false’ an area around the experimental plot is also modelled.
- Distance-move: the number of cells moved by adult flies each tick (day).
- Prob-feed: probability of finding and feeding on a host on any given day of the feeding cycle given one host present within a square neighbourhood of the fly of length 1 km (*σ* in ODE model).
- Days-to-starve: number of days it takes for a fly to starve.
- DD-coef: pupal density-dependent mortality coefficient.

*Global variables set during setup*

- Number-hosts: tracks the numbers of hosts over time.
- Larv-dur: duration of the larval period in days.
- Pre-adult-mort: daily probability of density-independent mortality for pupae.
- Pupal-period: duration of the pupal period in days.
- Background-mortality: daily probability of density-independent adult death, independent from starvation.
- Pnf-lambda: probability of not feeding given lambda hosts (see ODE model description).
- Number-flies: records the number of adult flies present within the experimental plot.

*Patches*

Patches have a location state variable. In both the closed and open model scenarios, patches within the experimental plot subject to host decline have location 0. In the open model scenario, there are 2160 patches forming the experimental plot with location 0, between y coordinates 28 to 53 and x coordinates 26 to 117. There are 2680 patches with location 1 surrounding the experimental plot. These are located below the experimental plot between y 18 to 28 and x 26 to 117; above the experimental plot between y 53 to 63 and x 26 to 117; to the left between y 18 to 63 and x 16 to 26; and to the right y 18 to 63 and x 117 to 127. There are 7160 remaining patches around the outside with location 2 (Fig A). The proportion of pupae that die each day from density-dependent mortality are calculated separately for each of these areas, as described below.

**Fig. A.** **Division of model grid into three zones for implementing host decline and density-dependent mortality.** Locations of patches within the experimental plot (location 0) – white, in a buffer zone around the outside of the experimental plot (location 1) – blue, and remaining patches (location 2) – green.


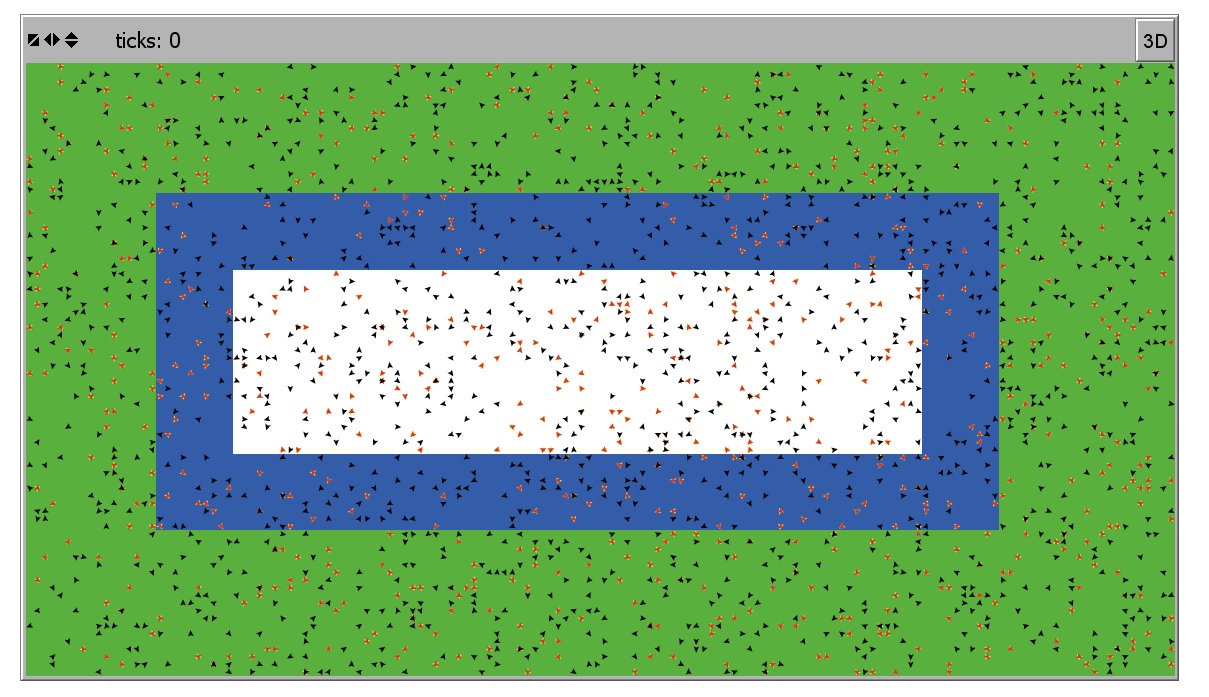


*Agents*

There are three types of agent - adult female flies, adult male flies and pupae. Adult male flies do not have any state variables. Adult female flies track the number of days since last larviposition using the state variable days-since-larv. Pupae have an age (days) state variable.

*Setup*

During ‘setup’ the spatial grid is created and the values for the global variables are set. Starting agents are also created during setup. Initial agents are placed at random spatial locations at the start of model runs.

As per the ODE, for the scenario without fly movement into and out of the experimental plot, the starting numbers were 250 pupae and 250 adults – scaled from some unknown true density. This is equivalent to a scale pupal density of approximately 0.46 per km^2^. For the open-system scenario, comprising 3000 km^2^, we calculated the starting numbers by multiplying this total area by the scaled density giving 1830 adults and 1830 pupae.

*Process overview and scheduling*

There are seven procedures within the model that occur every time-step (day):

1. Host-decline
Calculates the number of hosts remaining inside the Nagupande experimental plot (patches with location 0) and stores the result in a list - number-hosts. If closed-system = true, then the host density outside the experiment plot (patches with location 1 and 2) is assumed to remain equivalent to the host density within the experimental plot before the start of the experiment. The number of hosts within the experimental plot at each time step is calculated using Eq. 2, with parameter estimates obtained from model fits to the number of hosts shot each month, as described in the main text of the manuscript.

2. Starvation
Calculates the starvation mortality rate (Eq. 5, 6), using the density of hosts calculated in the host-decline procedure for inside the experimental plot and the density of hosts at the start of the experiment for outside the experimental plot. Rates then converted into a daily probability of mortality. Individual adult female and male flies select a random number between 0 and 1, if the number is less than the probability of mortality for that day in that location, they die.

3. Activity
Individual adult female and male flies select a random heading between 0 and 360^o^. They then move a straight-line distance in that direction, the distance is set on the interface via the slider ‘distance-move’. If the closed-system scenario is being modelled, this movement does not affect model output.

4. Reproduce
Female flies are assumed to be inseminated upon emergence. They produce larvae every ‘larv-dur’ days. Each female records how long since she last deposited a larva through the variable ‘days-since-larv’. Due to the relatively short larval period compared with pupal and adult stages, larvae are not modelled explicitly. Larvae are deposited as pupae which then, with equal probability, either change into adult males or females after pupal-period days. The state variable ‘age’ tracks the age of individual pupae.

5. Pupal-death
Pupae are subject to a daily probability of density-independent mortality (pre-adult-mort). Individual pupae select a random number between 0 and 1, if this is less than pre-adult-mort, they die.

Pupae are also subject to density-dependent mortality. This density-dependent mortality has to be spatially explicit. We therefore assume that the observed average monthly numbers of tsetse caught is proportional to the total numbers of tsetse present in the experimental plot. The density-dependent mortality coefficient (*δ* Table 1) is therefore assumed to be per 540 km^2^. If the closed-system scenario is being modelled, the total number of pupae present is multiplied by the density-dependent mortality coefficient to calculate the density-dependent mortality rate. This is converted to a daily probability. The probability of density-dependent mortality is then multiplied by the total number of pupae present, to calculate how many die.

If the open-system scenario is being modelled, the mortality due to density-dependence will be higher in areas furthest away from the experimental plot where the population has reduced mortality from starvation. This needs to be accounted for when calculating density-dependent mortality. To do this the entire area is split into three zones using the patch location variable (Fig. S3.A).

Pupae on patches with location 0 – inside the experimental plot – are subject to density-dependent mortality as described above for the closed system. For pupae in locations 1 and 2, the number of pupae subject to density-dependent mortality is scaled by 1.24 (2680/2160) and 3.31 (7160/2160) respectively to account for the different sizes of these two areas from the experimental plot.

6. Adult-death
Calculates the probability of mortality for adult female and male flies from other causes including senescence and predation. Each individual selects a random number between 0 and 1, if this is less than background-mortality then they die.

7. Record-flies
This is a reporter- reports the total number of flies over time in the experimental plot.
