# Supplementary material for: Host-seeking efficiency can explain population dynamics of the tsetse fly Glossina morsitans morsitans in response to host density decline
Source: PLoS Negl Trop Dis. 2017 Jul 3;11(7):e0005730. doi: 10.1371/journal.pntd.0005730 (PMC5510883; doi:10.1371/journal.pntd.0005730)
Supplement: S4 File — (DOCX) [file pntd.0005730.s004.docx]

**Supplementary File 4. Effect of host growth rate on estimated numbers of hosts during Nagupande experiment according to model of host kills over time (excluding the first two months).** Using data from all months, the fitted parameters *k_1_* = 2591, *k_2_* = 0.0794, were used to calculate the number of hosts over time assuming a growth rate of zero (solid line) or 0.015 month^-1^ (dashed line).

**
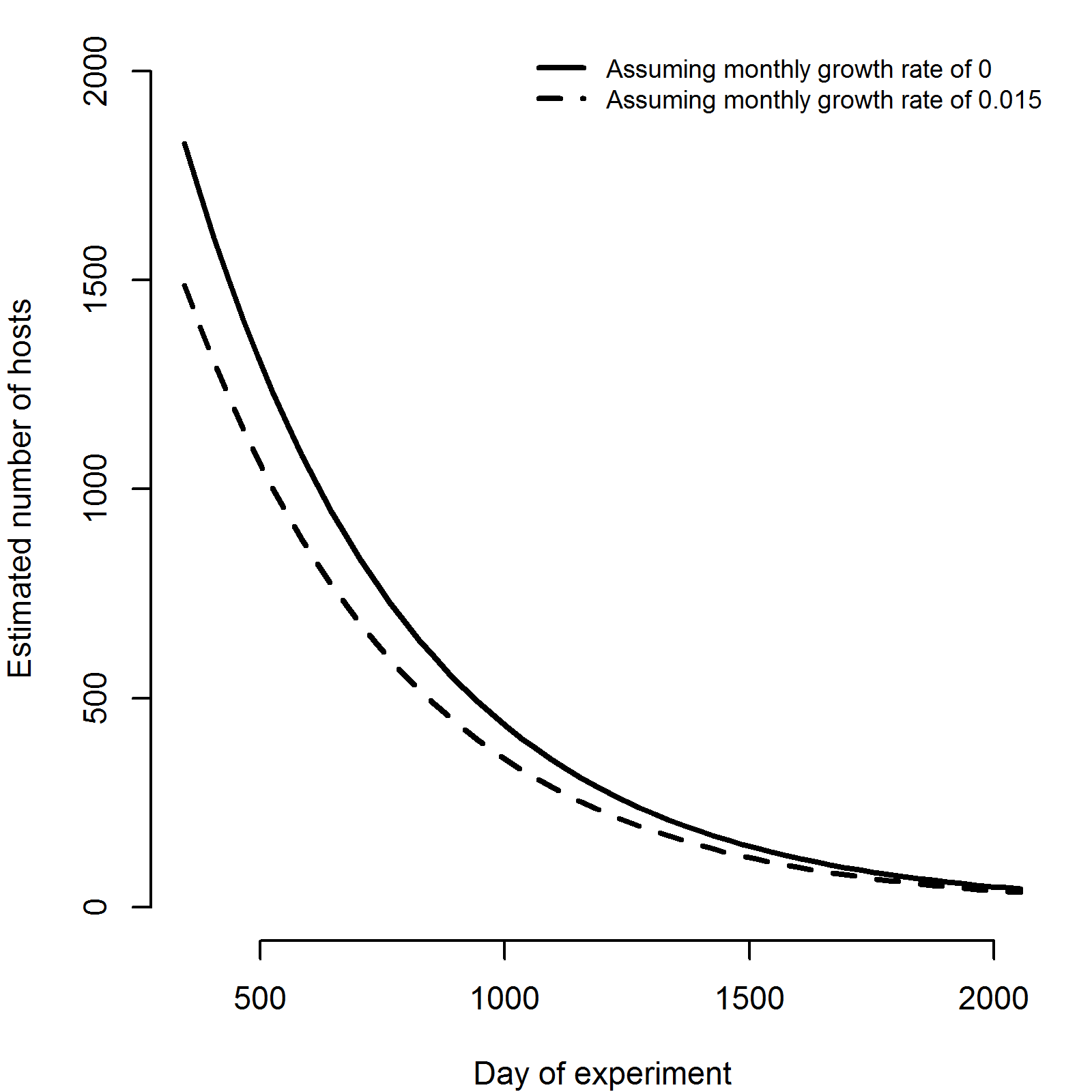
**
